# Self-reported drunkenness among adolescents in four sub-Saharan African countries: associations with adverse childhood experiences

**DOI:** 10.1186/1753-2000-4-17

**Published:** 2010-06-22

**Authors:** Caroline W Kabiru, Donatien Beguy, Joanna Crichton, Alex C Ezeh

**Affiliations:** 1 African Population and Health Research Center (APHRC), 2nd Floor Shelter Afrique Centre, P. O. Box 10787-00100, Nairobi, Kenya

## Abstract

**Background:**

Consumption of alcohol is associated with acute and chronic adverse health outcomes. There is a paucity of studies that explore the determinants of alcohol use among adolescents in sub-Saharan Africa and, in particular, that examine the effects of adverse childhood experiences on alcohol use.

**Methods:**

The paper draws on nationally-representative data from 9,819 adolescents aged 12-19 years from Burkina Faso, Ghana, Malawi, and Uganda. Logistic regression models were employed to identify correlates of self-reported past-year drunkenness. Exposure to four adverse childhood experiences comprised the primary independent variables: living in a food-insecure household, living with a problem drinker, having been physically abused, and having been coerced into having sex. We controlled for age, religiosity, current schooling status, the household head's sex, living arrangements, place of residence, marital status, and country of survey. All analyses were conducted separately for males and females.

**Results:**

At the bivariate level, all independent variables (except for coerced sex among males) were associated with the outcome variable. Overall, 9% of adolescents reported that they had been drunk in the 12 months preceding the survey. In general, respondents who had experienced an adverse event during childhood were more likely to report drunkenness. In the multivariate analysis, only two adverse childhood events emerged as significant predictors of self-reported past-year drunkenness among males: living in a household with a problem drinker before age 10, and being physically abused before age 10. For females, exposure to family-alcoholism, experience of physical abuse, and coerced sex increased the likelihood of reporting drunkenness in the last 12 months. The association between adverse events and reported drunkenness was more pronounced for females. For both males and females there was a graded relationship between the number of adverse events experienced and the proportion reporting drunkenness.

**Conclusions:**

We find an association between experience of adverse childhood events and drunkenness among adolescents in four sub-Saharan African countries. The complex impacts of adverse childhood experiences on young people's development and behavior may have an important bearing on the effectiveness of interventions geared at reducing alcohol dependence among the youth.

## Background

Consumption of alcohol is associated with acute and chronic adverse health outcomes including cardiovascular diseases, liver damage, cancers, psychiatric conditions, as well as intentional and unintentional injuries [1]. Besides direct health risks, alcohol consumption is also correlated with negative social and behavioral outcomes, such as risky sexual behavior [2-4]. Despite widespread interventions to raise awareness of the harmful consequences of alcohol use, global data suggest an increase in alcohol consumption among young people [1,5]. For example, data from the 1998 National Drug Strategy Household survey in Australia show that successive birth cohorts were more likely to report alcohol use by age 15, with 16% of adults born between 1940-1944 reporting such use compared to 56% of those born in 1980-1984 [6]. In the United States, an analysis of national survey data collected between 1979 and 2005 shows a considerable increase in volume of alcohol consumed and the number of days in which respondents report that 5 or more drinks were consumed among young people aged 18-25 [7].

Concern about alcohol consumption among young people has led to a mushrooming of literature that seeks to understand the correlates of alcohol abuse among the youth. Although there is now ample literature on alcohol consumption in sub-Saharan Africa, much of it focuses on the links between alcohol consumption and sexual behavior and sexually transmitted diseases (in particular HIV) among adult and adolescent populations [2,4,8-10]. There is, however, a paucity of studies that explore the determinants of alcohol use among adolescents in sub-Saharan Africa and, in particular, that examine the effects of adverse childhood experiences on alcohol use. Yet, the complex impacts of adverse childhood experiences on young people's development and behavior may have an important bearing on the effectiveness of interventions geared at reducing alcohol consumption among the youth. With this in mind, we examine the association between four adverse childhood experiences (i.e. living in a food insecure household, living in a household that suffered due to an adult member's drinking, having been physically abused, and having been coerced into having sex) and self-reported past-year drunkenness among adolescents aged 12-19 years living in Burkina Faso, Ghana, Malawi, and Uganda.

### Alcohol consumption among adolescents and youth in sub-Saharan Africa

Existing literature on alcohol consumption among adolescents in sub-Saharan Africa suggests that a substantial proportion of adolescents have consumed or currently consume alcohol. Two Ghanaian studies conducted among secondary school students [8] and among nationally-representative samples of in- and out-of-school youth [9] found that the prevalence of lifetime alcohol use was approximately 25%. According to the 2003 World Health Survey [1], the proportion of 18-24 year old males reporting heavy drinking (defined as consuming five or more standard drinks in one sitting at least once a week) was estimated at 8% in Burkina Faso, 1% in Ghana, and 5% in Malawi. Comparative figures for females were 5%, 0.3%, and 0.2% in Burkina Faso, Ghana, and Malawi respectively. Age-disaggregated data were not available for Uganda in the World Health Survey; however, data from the 2003 Ugandan Global School-based Student Health Survey show that 14% and 12% of boys and girls aged 13-15 years, respectively, reported that they had ever drunk so much alcohol that there were really drunk [10]. In a study conducted among school going adolescents aged 11-17 years in Uganda, 18% of adolescents reported that they had ever drank alcohol [11]. Studies conducted elsewhere in sub-Saharan Africa, also show that a relatively high proportion of young people report alcohol use. For example, in a study among secondary schools students in south western Nigeria [12], 13% of students reported current alcohol use while 26% had ever consumed alcohol.

Although cross-national variations in the measures and approaches used to assess alcohol use make it difficult to make comparisons across countries, existing data suggest that adolescents in sub-Saharan Africa consume less alcohol than their peers in North America and Europe [1,5]. Further, global data show that the disease burden, as measured by Disability Adjusted Life Years (DALYs), attributable to alcohol use is significantly higher in Europe and the Americas. However, within Africa, the overall disease burden attributable to alcohol use is not insignificant and evidence suggests that alcohol-related problems will contribute more to the overall disease burden over time [13,14]. Variations in socio-cultural contexts, as well as policies relating to alcohol production, sales and consumption may also lead to wide diversity in alcohol consumption patterns within the region [14]. For example, in Malawi and Uganda, the sale of alcoholic beverages to children under the age of 18 years is prohibited, while Burkina Faso and Ghana have no age limits for the purchase of alcohol. Further, while Uganda has no restrictions on the hours of sale, days of sale, or places where alcohol can be sold, Burkina Faso has some restriction on where alcohol can be sold [15]. Alcoholic beverages in all the four countries are taxed [15]; however, taxes are primarily applied to industrially-produced alcoholic beverages and not to traditional home-made brews, which are readily available and cheaper in many sub-Saharan African countries.

### Adverse childhood experiences and consequences

A substantial body of literature based on data collected outside of sub-Saharan Africa points to the long-term consequences of adverse childhood experiences. For example, in addition to the inherent trauma and rights violations involved in child sexual abuse, a history of childhood sexual abuse has been shown to be associated with depression and other psychosocial impacts that increase the likelihood of risky behavior later in life [16-18]. Sexual abuse has been linked to teenage drug and alcohol use, younger age at first intercourse, and teenage parenthood [19-23]. Dube and colleagues [24] postulate that physiologic changes in response to abuse and trauma in childhood may impact on neurodevelopment in ways that hinder emotional coping, leading to potentially harmful behavior such as substance use and dependence.

Sexual victimization of children is not uncommon in sub-Saharan Africa. In a study conducted among adolescent females in Rakai, Uganda, 14% of respondents reported that their first sexual intercourse was forced [20]. Lalor [25] in his review of the literature on sexual abuse in the region found that between 3% and 7% of respondents in several South African studies reported unwanted or forced sexual intercourse before the age of 17 or 18, with the proportions rising to between 26% and 54% when unwanted kissing and touching were considered. In the few studies in Lalor's review that examined male-female differences, a greater proportion of females than males reported sexual abuse.

Food insufficiency is a common problem in resource-constrained households. Low incomes and high food prices, especially in the context of a global economic crisis, mean that the poor in many nations, and in particular those in the developing world, have to reduce food intake and rely on less-nutritious foods [26]. With the exception of a few studies examining the link between food insecurity and sexual risk behavior among adults [27], there is, to the best of our knowledge, no study documenting the association between food insufficiency and behavioral as well as psychological outcomes in children and adolescents in sub-Saharan African countries. Studies from the United States show that adolescents from food insecure household are more likely to have or to report chronic depression [28], suicidal attempts [28], desire to die [28], thoughts of death [28], irritability, anxiety or worry [29], socialization problems such as aggression [30], and poorer schooling outcomes [30,31]. The processes that lead to the observed linkages between food insecurity and behavioral and psychosocial outcomes in children are not well understood. Alaimo and colleagues [28] postulate biological mechanisms, stressor effects, and indirect associations through modified parental emotions and parenting behavior. With respect to modified parenting behavior, they suggest that in food insufficient households, parents may be subjected to high levels of stress and consequently be unable to optimally care for their children. As a result of poor parental control, children from food insecure households may be more likely to engage in risk behavior including alcohol use.

Growing up in a household where a parent or other household member has a drinking or drug problem that negatively impacts on the entire household may also contribute to behavioral problems during adolescence [32,33]. Zucker and colleagues [34] in their review of the literature on early developmental influences of underage and problem drinking highlight several possible pathways through which familial alcoholism may lead to drinking problems in children and adolescents. First, young people's attitudes towards alcohol are shaped in part by interactions in the social context in which children are raised. Second, having parents or other adults who drink in a household increases the likelihood that alcohol is available and accessible in the home. Third, if the adult with problem drinking is a primary caregiver, this may have implications for parenting behavior and levels of discipline. Last, genetic predisposition to problem drinking is likely where the child and adult with problem drinking are biologically related.

Understanding the long-term consequences of adverse childhood experiences on alcohol abuse among adolescents is useful for informing alcohol abuse prevention and treatment programs. Yet, there is a paucity of research on adverse childhood experiences and later alcohol use in sub-Saharan Africa outside of South Africa. Further, with few exceptions [35], there is a dearth of studies examining the effects of exposure to multiple adverse events during childhood on alcohol abuse. Given that youth comprise a significant proportion of sub-Saharan Africa's population and since young people's behavior have critical long term implications for a healthy and successful transition to adulthood [36], this study seeks to address these gaps by drawing on a rich set of nationally-representative data collected from adolescents aged 12-19 years living in Burkina Faso, Ghana, Malawi, and Uganda. We hypothesize that young people who have experienced adverse events during childhood will be more likely to report drunkenness in the 12 months preceding the survey. Further, we postulate that exposure to multiple adverse events heightens the likelihood of reporting being drunk.

## Methods

### Study Sample and Design

The present study is based on secondary analyses of nationally-representative data collected from adolescents aged 12-19 years as part of the multi-year *Protecting the Next Generation: Understanding HIV Risk among Youth *(PNG) study conducted in Burkina Faso, Ghana, Malawi, and Uganda by the Guttmacher Institute, the African Population & Health Research Center and their partners in each of the four countries. The paper draws on data from 9,819 adolescents aged 12-19 years from Burkina Faso Ghana, Malawi, and Uganda. Overall, data were collected from 5,955 respondents in Burkina Faso, 4,430 in Ghana, 4,031 in Malawi, and 5,112 in Uganda. Due to the sensitive nature of questions about physical abuse, these questions were administered to a smaller sub-set and were only asked if there was no one over three years of age within listening distance. The number of adolescents who responded to these questions was 10,487 respondents. Out of this subset, we restrict the analyses to respondents with complete information on the four adverse effects (N = 9,819 or 93.6%). The data are therefore weighted to adjust for the sample design, household and individual non-response, as well as the sub-sampling of only one eligible adolescent per household for the portion of the questionnaire with the physical abuse questions.

### Informed Consent and Ethical Clearance

Informed consent was sought from each adolescent prior to conducting the interview. For adolescents aged 12-17 years, parental/guardian consent was obtained prior to seeking consent from the adolescent. Ethical approval for the study was granted by: Comité National d'Éthique pour la Recherche en Santé (Burkina Faso), the University of Ghana Medical School Institutional Review Board (IRB), the National Health Sciences Research Committee for Ethical Approval in Malawi, the Uganda National Council for Science and Technology, and the Guttmacher Institute's IRB (United States). Detailed descriptions of the study sample and methodology for the larger study are provided elsewhere [37-40].

### Measures

Outcome variable: The primary outcome variable was whether or not the respondent self-reported past-year drunkenness. This variable was derived from a single question: "In the last 12 months, have you ever gotten "drunk" from drinking alcohol-containing beverages?"

Our main explanatory variables were respondents' experience of four adverse events in childhood: Having lived in a household where there was not enough food for everyone; living in a household that suffered because of a household member's heavy drinking; experience of physical abuse; and having been coerced into having first sex. Participants' exposure to these stressors was assessed through the following four items in the interview questionnaire: "Think now about what your family life was like up until age 10, how often did your family not have enough food to feed everyone?" (response categories: very often, somewhat often, not often at all, or never); "When you were growing up until age 10, did your household suffer because someone drank too much alcohol?" (response categories: yes or no); "When you were growing up until age 10, did a parent or other adult living in your home ever hit you hard enough to leave marks or cause injury?" (response categories: yes or no); and "Thinking about the first time you had sexual intercourse, would you say you were very willing, somewhat willing or not willing at all?" The United Nations defines a child as anyone below the age of 18 years [41]. Thus, to ensure that we were capturing coerced first sex that occurred during childhood and that coerced sex preceded reported drunkenness in the last year, a respondent was considered to have had coerced first sex if he or she reported that first sex occurred 2 or more years prior to the survey (when the oldest respondent would have been 17 years) and that he or she was not willing at all to have sex then.

We controlled for variables that have been found to be associated with alcohol use and other risk behaviors among adolescents [12,24,34]. These included participants' reported age; current schooling status; gender of the head of household; respondents' living arrangements (response categories: living with both parents, with mother only, with father only, or with neither parent); rural or urban residence; marital status (response categories: ever married or never married); religiosity; and country of residence. Religiosity was derived from a single question asking "How important is religion in your life?" Responses were coded into three categories: very important, somewhat important, and not important or does not have a religious affiliation.

### Analyses

Univariate statistics were computed to describe the respondents' social and demographic characteristics, as well as reported adverse experiences and alcohol use. Bivariate and univariate statistics were computed using PASW software, Version 17.0 [42]. Logistic regression models were employed to identify correlates of self-reported past-year drunkenness while controlling for age, religiosity, current schooling status, the household head's sex, living arrangements, place of residence, marital status, and country of survey. All analyses were conducted separately for males and females. We conducted the computations for the logistic regression in Stata, Version 10.1 [43].

### Participants

Background characteristics of respondents are summarized in Table 1. Males comprised 51% of the sample. The majority of respondents (62%) were enrolled in school with a greater proportion of males (66%) than females (58%) being in school. About three-quarters (76%) of the respondents were living in male-headed households. Just under half of the adolescents (47%) were living with both parents with a greater proportion of males (50%) than females (43%) reporting that they lived with both parents. Only 6% of respondents were living with their biological father only. About a quarter of respondents (26%) lived in urban areas. Only 6% of respondents had ever been married. However, a greater proportion of females (11%) than males (1%) had ever been married. The sample was almost equally split by country though a slightly higher proportion of respondents were from Burkina Faso (29%). The majority of respondents (82%) viewed religion as 'very important' in their lives.

**Table 1:** Respondents' sociodemographic characteristics and exposure to adverse childhood experiences

	Male	Female	Total
	n = 4,968 (51%)	n = 4,851 (49%)	N = 9,819 (100%)
*Sociodemographic characteristics*			
Mean age in years (standard deviation)	15.0 (2.19)	15.1 (2.23)	15.0 (2.21)
% In school	65.7	58.3	62.1
% Male-headed household	78.1	74.5	76.3
Living arrangements			
Both parents ^a^	50.0	43.2	46.7
Mother only ^a^	16.7	15.8	16.3
Father only ^a^	7.3	5.3	6.3
Neither parent ^a^	26.0	35.7	30.8
% Urban residence	24.5	28.4	26.4
% Ever married	1.3	10.7	5.9
Country of residence			
Burkina Faso ^a^	28.6	28.9	28.7
Ghana ^a^	24.2	25.7	24.9
Malawi ^a^	23.8	22.7	23.2
Uganda ^a^	23.4	22.8	23.1
Importance of religion in life			
Very important ^a^	81.5	82.3	81.9
Somewhat important ^a^	13.1	13.0	13.1
Not important/no religion ^a^	5.4	4.7	5.0
*Exposure to adverse childhood experiences*			
Frequency with which family did not have enough food before respondent was 10 years			
Very often ^a^	11.1	10.3	10.7
Somewhat often ^a^	25.5	25.7	25.6
Not often at all/Never ^a^	63.4	64.0	63.7
% of respondents who lived in a household with a problem drinker before age 10 years	18.5	20.7	19.6
% of respondents who were physically abused before age 10	20.3	15.8	18.0
% of respondents whose first sex was coerced	1.8	5.6	3.7

Sample sizes are weighted

^a ^% of total sample

## Results

Table 1 also summarizes the prevalence of adverse childhood experiences. Eleven percent of respondents stated that up to the age of 10, their family did not have enough food 'often' while 26% stated that this occurred 'somewhat often'. A fifth (20%) of respondents had lived in a household with a problem drinker before age 10. Physical abuse before age 10 was reported by 18% of respondents with more males (20%) than females (16%) reporting so. Four percent of all respondents and 15% of sexually experienced respondents (not shown in the table) reported that their first sex occurred before age 18 and was coerced. More females (6%) than males (2%) reported that they had been coerced into having their first sexual intercourse. Proportions of those coerced into first sex are higher when we consider data from those who are sexually experienced - 23% of females and 7% of males (not shown in the table).

Table 2 summarizes the bivariate analyses between self-reported past-year drunkenness and independent and control variables. Seven percent of respondents reported that they had been drunk or intoxicated in the 12 months preceding the survey. Consistent with prior research in sub-Saharan Africa showing that a greater proportion of males report alcohol use [44-46], males were significantly more likely to report drunkenness (9% among males versus 5% among females). At the bivariate level, all the independent variables (with the exception of coerced sex among males) were associated with the outcome variable at the 0.05 level of statistical significance. While 9% of respondents who reported frequent food shortages reported being drunk, just under 7% of those who reported infrequent or no food shortages reported being drunk in the 12 month period preceding the survey. Fourteen percent of respondents who had lived in a household with a problem drinker reported being drunk compared to 6% of those who were not exposed to this adverse event. Twice as many respondents reporting physical abuse in childhood (12%) reported that they had been drunk compared to those reporting no physical abuse (6%). A greater proportion of respondents who had been coerced into their first sexual intercourse (10%) reported that they had been drunk compared to 7% of their counterparts who did not report coerced first sex.

**Table 2:** Percentage of respondents reporting drunkenness in the last 12 months by sociodemographic characteristics and exposure to adverse childhood experiences (N = 9,819)

	Male n = 4,968	***p*-value**^**a**^	Female n = 4,851	***p*-value**^**a**^	Total N = 9,819	***p*-value**^**a**^
	%		%		%	
Self-reported past-year drunkenness ^b^	9.4		5.0		7.2	
*Exposure to adverse childhood experiences*						
Frequency with which family did not have enough food before respondent was 10 years						
Very often	10.7	*	7.0	*	8.9	**
Somewhat often	10.8		5.5		8.1	
Not often at all/Never	8.6		4.4		6.5	
Did respondent live in a household with a problem drinker before age 10 years						
No	7.5	**	3.4	**	5.5	**
Yes	17.8		11.1		14.3	
Was respondent physically abused before age 10 years						
No	8.4	**	4.0	**	6.2	**
Yes	13.5		9.9		12.0	
Was respondent coerced into first sex before age 18 years						
No	9.3		4.7	**	7.1	*
Yes	12.6		9.5		10.2	
*Sociodemographic characteristics*						
Importance of religion in life						
Very important	9.2		5.0		7.1	
Somewhat important	11.5		4.8		8.2	
Not important/no religion	7.9		5.3		6.7	
Schooling status						
In school	7.7	**	4.0	**	6.0	**
Out of school	12.6		6.3		9.2	
Sex of household head						
Male	9.2		4.7		7.0	
Female	9.9		5.8		7.7	
Living arrangements						
Both parents	8.2	**	4.6		6.5	
Mother only	10.6		5.2		8.0	
Father only	8.3		5.9		7.3	
Neither parent	11.4		5.2		7.8	
Area of residence						
Urban	11.2	*	4.1		7.4	
Rural	8.8		5.3		7.1	
Marital status						
Never married	9.1	**	4.7	*	7.0	**
Ever married	31.8		7.4		10.1	
Country of residence						
Burkina Faso	5.3	**	3.1	**	4.3	**
Ghana	11.3		6.5		8.9	
Malawi	7.6		1.9		4.9	
Uganda	14.2		8.5		11.4	

Sample sizes are weighted

^a^*p*-values show the levels of significance of the association between each socio-demographic variable and self-reported past-year drunkenness by gender and for the total sample (e.g. 8% of in-school males report drunkenness while 13% of out-of school males do so. These proportions are statistically significantly different)

^b^Gender difference is statistically significant at the .05 level of significance

*p < .05; ** p < .01

Among the control variables, respondents' age, sex, schooling status, marital status, and country of residence were significantly associated with self-reported past-year drunkenness when male and female data were combined. Among both males and females, respondents who reported being drunk were older (males 16.1 years, SD = 2.16; females 15.6 years, SD = 2.40) than those reporting that they were not drunk at any time in the preceding 12 months (males 14.9 years, SD = 2.16; females 15.0 years, SD = 2.22) (results not shown in the tables). Overall, male and female respondents who were out of school were more likely to report drunkenness than those in school. Ten percent of respondents who had ever been married reported drunkenness compared to 7% of never married respondents. Close to three times as many Ugandans (11%) as Burkinabés (4%) reported that they had been drunk. Among males, living arrangements and area of residence were also significantly associated with reported drunkenness. Approximately 8% of male respondents living with both parents or with fathers only reported being drunk in the last 12 months, compared to 11% among those living with only their mother or neither parent. With respect to area of residence, a greater proportion of males living in urban areas (11%) reported being drunk in the preceding 12 months than those living in rural areas (9%).

Table 3 presents two sequential models to assess the net effects of the adverse childhood events when controlling for sociodemographic characteristics. The first model includes only the main explanatory variables while the second adds the control variables. The food insecurity measure was not associated with reported drunkenness for both males and females. This suggests that our measure of food insecurity may be relatively weak or that the pathways to alcohol use are more indirect than for other adverse experiences.

**Table 3:** Adjusted odds ratio estimates of self-reported past-year drunkenness in the last 12 months, by gender

	Male				Female			
	
	OR	95% CI for OR	OR	95% CI for OR	OR	95% CI for OR	OR	95% CI for OR
*Adverse childhood experiences*								
Frequency of food shortage (ref. not often/never)								
Very often	0.98	[0.68,1.41]	0.81	[0.54,1.23]	1.2	[0.73,1.95]	1.25	[0.74,2.11]
Somewhat often	1.12	[0.84,1.48]	1.01	[0.74,1.38]	0.98	[0.64,1.50]	0.97	[0.64,1.49]
Lived in a house with an alcoholic before age 10 years	2.54**	[1.95,3.31]	2.33**	[1.74,3.11]	3.07**	[2.18,4.32]	2.68**	[1.89,3.80]
Physically abused before age 10 years	1.53**	[1.17,2.00]	1.49**	[1.13,1.97]	2.08**	[1.44,3.00]	1.94**	[1.35,2.80]
Coerced into having first sex	1.26	[0.50,3.16]	1.03	[0.39,2.72]	1.91*	[1.14,3.20]	1.67^†^	[0.93,3.00]
*Sociodemographics*								
Age			1.24**	[1.17,1.32]			1.03	[0.93,1.13]
Importance of religion (ref. very important)								
Somewhat important			1.51*	[1.05,2.18]			0.93	[0.57,1.51]
Not important/no religion			1.35	[0.79,2.33]			1.33	[0.67,2.65]
In school (ref. out of school)			0.56**	[0.41,0.77]			0.51**	[0.33,0.78]
Female-headed household (ref. male-headed household)			0.8	[0.54,1.18]			1.31	[0.87,1.97]
Living arrangements (ref. both parents)								
Mother only			1.18	[0.77,1.83]			0.75	[0.44,1.28]
Father only			0.82	[0.53,1.26]			0.92	[0.39,2.19]
Neither parent			1.07	[0.77,1.49]			0.79	[0.49,1.28]
Rural resident (ref. urban resident)			0.68^†^	[0.47,1.00]			1.26	[0.81,1.95]
Ever married (ref. never married)			1.78	[0.77,4.13]			1.01	[0.54,1.89]
Country of survey (ref. Burkina Faso)								
Ghana			2.41**	[1.52,3.81]			2.35**	[1.47,3.76]
Malawi			2.00*	[1.17,3.41]			0.69	[0.28,1.70]
Uganda			3.73**	[2.44,5.70]			2.81**	[1.70,4.63]
Constant	0.07**	[0.06,0.09]	0.00**	[0.00,0.02]	0.03**	[0.02,0.04]	0.01**	[0.00,0.07]
N	4,968		4,943		4,851		4,832	

Sample sizes are weighted and coefficients provided are odds ratios

^†^p < 0.10; *p < .05; ** p < .01

For males, only two adverse childhood events emerged as significant predictors of self-reported past-year drunkenness: living in a household with a problem drinker before age 10 and being physically abused before age 10. The associations remained significant after control variables were added to the model. As at the bivariate level, age and schooling status were significantly associated with the outcome measure. Compared with respondents who are out of school, males who were in school were less likely to report drunkenness. Religiosity also emerged to be a significant predictor of drunkenness among males. Specifically, respondents who reported that religion was somewhat important were significantly more likely to report being drunk than their peers who reported that religion was very important in their lives. This is also consistent with previous work in Lebanon and the United States [47,48] showing the protective nature of religiosity.

The association between adverse childhood experiences was more pronounced for females. Exposure to family-alcoholism and experience of physical abuse or coerced sex were associated with an increased likelihood of reporting drunkenness in the last 12 months. After adding the control variables, the association between coerced sex and reported drunkenness was only marginally significant. Contrary to what was observed for boys, age, religiosity, living arrangements, and marital status were not significantly associated with drunkenness among females. As with males, being enrolled in school was associated with a lower likelihood of reported drunkenness for females. Given high levels of unemployment in these countries, most young people who are out-of-school are either jobless or are forced to take up low-paying informal jobs. Consequently, being out of school may involve a lack of activities, income, and structure that predisposes adolescents to substance use.

Among both males and females, we find that Burkinabés were significantly less likely to report drunkenness compared to respondents from other countries (except for Malawian females). Socio-cultural factors such as religious affiliation may drive these cross-national variations in drinking patterns. Burkina Faso is the only one of the four countries studied that has a predominately Muslim population. Thus, while there is no legal age restriction for the purchase of alcohol in Burkina Faso, religious proscriptions may affect alcohol consumption patterns among Burkinabé youth.

**Figure 1:**
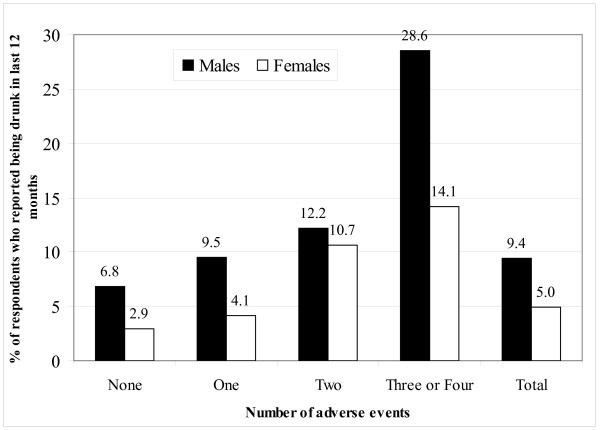
**Percentage of respondents reporting drunkenness in last 12 months, by gender**.

To examine the relation between the number of adverse experiences and self-reported past-year drunkenness, we generated a variable indicating the total number of adverse events each respondent reported. For food insufficiency, we considered a respondent to have experienced the event if they responded that they had experienced food shortages 'somewhat often' or 'very often.' The combined adverse events scores ranged from 0 to 4. Because of the small number of respondents who had experienced all four events we combined this group with those who had experienced three events. We then compared the proportion of respondents reporting drunkenness in the preceding 12 months. As Figure 1 shows, for both males and females, there is a graded relationship between the number of events and the proportion reporting drunkenness. Ten percent of males and 4% of females who experienced one adverse childhood event reported drunkenness in the preceding 12 months whereas 29% and 14% of males and females, respectively, who experienced three or four adverse childhood events reported drunkenness.

## Discussion

Consumption of alcohol is associated with acute and chronic adverse health outcomes, as well as negative social and behavioral outcomes, such as risky sexual behavior. Despite widespread interventions to raise awareness of the harmful consequences of alcohol use, global data suggest an increase in alcohol consumption among young people. This study seeks to address the paucity of research exploring the determinants of alcohol use among adolescents in sub-Saharan Africa by examining the effects of adverse childhood experiences. We examined the association between four adverse childhood experiences (living in a food insecure household, living with a problem drinker, having been physically abused, and having been coerced into first sexual intercourse) and self-reported past-year drunkenness among adolescents aged 12-19 years living in Burkina Faso, Ghana, Malawi, and Uganda.

Waldrop et al [49] posit that "because of developmental processes still taking place in childhood, the trajectory involving alcohol use among persons with childhood trauma may involve greater behavioral and neurobiological consequences" (p. 441). Our study findings support evidence from other contexts that adverse childhood experiences can impact on young people's behavior. Consistent with previous research [3,21,23,50-52], we observe an association between reported physical (among both males and females) as well as sexual abuse (among females) and self-reported past-year drunkenness. Research conducted among a representative sample of current or former drinkers in the United States showed that respondents who reported childhood physical or sexual abuse, neglect, or alcohol abuse in the home were significantly more likely to report that they drank to cope compared to respondents who had not experienced these adverse events [52]. Association between childhood physical abuse and alcohol drinking has been evidenced in rural Taiwan [53] and South Korea [54]. Yen et al [53] hypothesize that alcohol consumption may help adolescents avoid recalling the episodes of childhood physical abuse or may be a manifestation of developmental psychopathology whereby "deficits in cognitive, social, emotional, and behavioral functioning associated with maltreatment" (p. 581) may predispose the adolescent to alcohol use in order to cope with these outcomes.

Our study also corroborates prior work [53,55,56] showing a link between household alcoholism and substance use. Growing up in a household that suffered due to a member's heavy drinking was associated with significantly greater odds of reporting alcohol use for both males and females. As stated earlier, familial alcoholism may increase the likelihood of alcohol use in adolescence through several pathways. First, family members with alcohol or drug problems may serve as behavioral models for young people living in the same household [32]. Second, family members suffering from alcohol dependence or other drug addictions may also store drugs and/or alcohol in the house making these substances more readily available to young people [32]. Third, familial alcoholism may be associated with family violence or parental neglect. Last, alcohol dependent parents may transmit to their adolescents genes that predispose them towards alcoholism [57].

With respect to our independent variables, we observed rates of coerced sex that mirror what has been observed in other studies in sub-Saharan Africa. For example, as stated previously, in his review of the literature on child sexual abuse in sub-Saharan Africa, Lalor [25] reported prevalence rates of forced oral, anal, or vaginal intercourse in South Africa ranging from 2% to 5% for males and from 3% to 6% for females. On the other hand, in their study among young females in Uganda, Koenig and colleagues [20], reported that 14% of respondents had been coerced into their first sexual intercourse. Yet, sexual coercion (especially if the perpetrator is known to the victim) is likely to be underreported in surveys [58] even where special measures are taken to ensure privacy and confidentiality.

Male-female comparisons of sexual abuse prevalence rates were similar to those found in previous studies [59-61], with more females than males stating that they had been sexually abused. The gender disparity in reporting sexual victimization may arise from underreporting of such incidents by males [60]. Conversely, greater disclosure of sexual abuse among females may, indeed, reflect greater sexual victimization of females because of increased vulnerability among female adolescents due to power inequalities related to socially constructed gender norms and practices [62,63], unequal access to resources [64] and age differences in relationships [65].

The findings of this study extend the knowledge on the impact of adverse childhood events on alcohol use among adolescents living in sub-Saharan Africa. These results should be interpreted in light of several study limitations. First, the cross-sectional study design precludes cause and effect inferences. However, while the outcome measure, drunkenness in the past 12 months, could reflect a behavior that started much earlier, the fact that the adverse experiences were limited to events before age 10 or at least two years prior to the survey suggests that the direction of the association is more plausible. Second, the reporting of sensitive information, especially socially-proscribed behaviors such as drinking among adolescents, may have influenced participants to provide socially-desirable responses despite measures taken to safeguard privacy and confidentiality of participants and their responses. Third, our outcome measure was based on the adolescents' response to a single question -- "In the last 12 months, have you ever gotten "drunk" from drinking alcohol-containing beverages?" The interpretation of the meaning of being drunk is highly subjective and likely shaped by the adolescents' own personal experiences and social context. Further, the measure does not include more objective measures of risky drinking such as the frequency or volume of alcohol consumption. Additional information on these measures would enable a more comprehensive measure of risky drinking. Although we rely on subjective appraisal of ever being drunk in the past year as a proxy for risky drinking [66], future research should incorporate multiple measures of risky drinking. Finally, we only had information on a limited number of adverse childhood experiences. Other studies conducted in the United States have found significant associations between alcohol use during adolescence and experience of emotional and physical neglect, as well as measures of household dysfunction such as parental discord, living with a mentally ill person, and living with someone who was imprisoned [24]. Future studies should incorporate more detailed measures on adversities in childhood in order to have a more nuanced understanding of effects of early-life adverse life experiences on alcohol use later in life. Notwithstanding these limitations, study findings suggest that adversities experienced during childhood may contribute to problem drinking among adolescents.

## Conclusions

Overall, our study findings corroborate previous research showing that adverse childhood experiences may predispose adolescents to alcohol use. Thus, early treatment for traumatic childhood experiences may be an essential component of interventions designed to prevent alcohol abuse among adolescents. Organizations working on alcohol abuse prevention and child abuse and neglect, respectively, should share experiences and take measures to coordinate efforts and services. In particular, African governments could play a greater role in developing more effective programs to prevent and address alcohol dependence. The finding that out-of-school young people were more likely to report drunkenness in the past 12 months indicates the need for alcohol abuse prevention interventions to target young people who are out of school as well as those in school. This study also draws attention to the importance of mental health, which has remained relatively obscure in health policies and programs, as well as research agendas, in sub-Saharan Africa. In particular, policies that mitigate the impacts of child abuse and maltreatment or help to prevent child abuse, as well as programs to help young people build the skills to avoid abusive relationships or to cope positively with traumatic events may also be useful in reducing alcohol abuse and associated negative outcomes.

The literature on adolescent alcohol use and child abuse and neglect is limited in the region, and therefore the phenomena are poorly understood. Future research studies need to include sufficiently detailed questions about substance use and childhood abuse and neglect (e.g. frequency, quantity, type of drugs). Further, we observe some evidence that the effects of alcoholism may be passed from generation to generation, thus there is need to study adult alcohol abuse in the region and its associations with adolescent alcohol use. Finally, there is a need for more qualitative studies to investigate youth culture and drinking in different African contexts, which could help bring a more nuanced approach to looking at the processes through which young people end up using and abusing substances.

## Competing interests

The authors declare that they have no competing interests.

## Authors' contribution

CWK conceptualized the manuscript idea, conducted the data analyses, participated in the literature review, and prepared the first draft of the manuscript. DB made substantive contributions to the conceptualization of the manuscript, contributed to the literature review and informed the data analyses. JC made substantive contributions to the conceptualization of the manuscript and contributed to the literature review. ACE made substantive contributions to the conceptualization of the manuscript. All authors read and approved the final manuscript
